# Role of the Interplay Between Autophagy and Cell Senescence in the Pathogenesis and Therapeutics of Glioblastoma in the Aging Population

**DOI:** 10.3390/cells14221764

**Published:** 2025-11-11

**Authors:** Eliezer Masliah

**Affiliations:** Department of Neurosciences, University of California San Diego, La Jolla, CA 92093, USA; emasliah@ucsd.edu

**Keywords:** glioblastoma, aging, cell senescence, autophagy, therapeutics

## Abstract

**Highlights:**

- Glioblastoma (GBM) is the most common malignant tumor of the brain in the aging population.
- The role of some of the aging hallmarks in the progression and suppression of GBM has been considered; however, the contribution of interactions among them has received less attention.
- For this review, we analyze the role of the interplay between cellular senescence and autophagy in the paradoxical effects on GBM tumor growth and suppression with the corresponding therapeutic implications.

**Abstract:**

Glioblastoma (GBM), formerly referred to as glioblastoma multiforme, represents the most prevalent and aggressive form of glioma, predominantly affecting the aging population. Despite considerable advances in recent years in elucidating its pathogenesis and developing novel immunotherapeutic approaches, the overall survival rate for patients with this central nervous system (CNS) neoplasm remains dismally low. Consequently, there is an urgent and unmet need to identify and characterize additional therapeutic targets that could be employed synergistically with existing treatment modalities to enhance both survival outcomes and quality of life. Among the emerging areas of investigation, substantial interest has been directed toward aging-associated molecular signaling mechanisms that also constitute key oncogenic pathways in GBM. These include aberrant growth factor signaling, hyperactivation of the PI3K/AKT/mTOR axis, and inactivation of critical tumor suppressor pathways such as p53 and retinoblastoma (RB). The dysregulation of these signaling cascades results in profound disturbances of essential cellular homeostatic processes, notably autophagy and cellular senescence, which are intimately involved in both tumor initiation and progression. This review aims to delineate the complex interplay between autophagy and cellular senescence within the context of aging-related GBM pathogenesis. Furthermore, it explores the relevant intracellular signaling transduction mechanisms that govern these processes and discusses prospective therapeutic strategies.

## 1. Introduction

Gliomas are a heterogeneous and sometimes mixed type of central nervous system (CNS) tumor that originates in glial cells, which support and protect neurons, their axons, and the cells lining the ventricular cavities [1], with high-grade gliomas representing the most malignant form. Considerable progress has been made in the last decade in developing novel experimental therapies for the most aggressive gliomas of the CNS. For instance, immunotherapeutic strategies targeting immune checkpoint blockade (e.g., PD-L1), chimeric antigen receptor T (CAR T) cell therapy, and vaccine therapy are showing new promise [2]. Moreover, current clinical studies are testing combinatorial therapies—including surgery, radiotherapy, and pharmacotherapy (e.g., temozolomide (TMZ))—with the aim of minimizing adverse side effects and augmenting antitumor immune responses [2].

**Figure 1:**
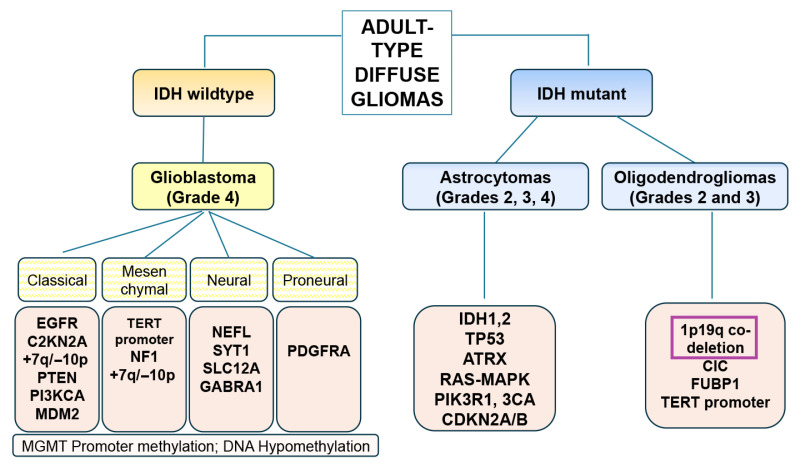
Diagrammatic representation of the classification of diffuse gliomas based on the WHO 2021 recommendations. These gliomas are divided into two groups depending on the presence or absence of IDH mutations. The IDH wildtype gliomas are high-grade glioblastomas, while the IDH mutant gliomas are astrocytomas (2–4) and oligodendrogliomas of lower grade (2–3). The purple box indicate the unique molecular genetic feature of this type of gliomas.

About one-third of all brain tumors are gliomas, accounting for nearly half of all primary brain tumors. In the aging population, the most common type of glioma is glioblastoma (GBM), formerly known as glioblastoma multiforme (Figure 1). The median survival time for patients with this aggressive form of glioma is approximately 15 months after diagnosis, despite a variety of treatments, including surgery, radiation, chemotherapy, and immunotherapy [3]. The neuropathological criteria for the classification of CNS tumors have changed considerably in recent years with the discovery of novel genetic and molecular markers [4,5]. The new 2021 WHO guidelines for the classification of CNS tumors involve the integration of molecular and neuropathological characteristics [5]. Also known as the WHO CNS5 classification, the main goals of this hybrid taxonomy were to achieve high reproducibility and simplify areas of redundancy or unclear nomenclature. Accordingly, gliomas are classified as adult-type diffuse gliomas, pediatric high-grade diffuse gliomas, pediatric low-grade diffuse gliomas, and circumscribed astrocytic gliomas. Adult-type diffuse gliomas are classified by IDH mutation status.

The IDH-wildtype subtype, which is the most aggressive form with a poor prognosis, is now referred to as glioblastoma, IDH-wildtype. Subtypes of GBM include the following: classical, mesenchymal, neural, and pro-neuronal (Figure 1). In contrast, IDH-mutant gliomas include astrocytomas and oligodendrogliomas. IDH mutations (specifically in IDH1 and IDH2) are genetic alterations that occur in a subset of these tumors, particularly those arising from lower-grade gliomas. These mutations are not present in primary GBMs [5]. IDH-mutant astrocytomas are now graded 2, 3, or 4 based on histological and molecular features. Importantly, a grade 4 tumor is no longer termed a glioblastoma but rather an astrocytoma, IDH-mutant, WHO CNS grade 4. IDH-mutant astrocytomas are more common in younger individuals and show slower progression. IDH mutations are also found in oligodendrogliomas (Figure 1). Tumors that, in addition to IDH mutations, display the 1p/19q codeletion are diagnosed as oligodendrogliomas [5] (Figure 1). The most frequent IDH mutation in gliomas is IDH1 p.R132H, accounting for around 90% of IDH-mutant cases.

**Figure 2:**
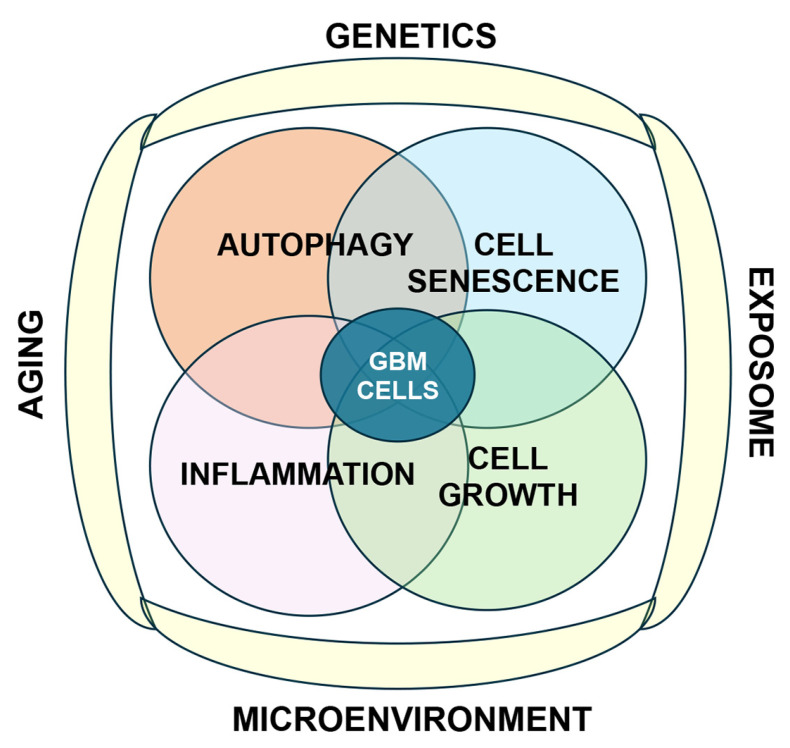
Conceptual depiction of the potential interactions between autophagy, senescence, inflammation, and cell growth in the pathogenesis of GBM. The interplay among these cellular mechanisms is modulated by environmental factors (exposome), the tumor microenvironment, the effects of the aging process, and the genetics of the host.

Another important feature for GBM classification is the methylation status of the O^6^-methylguanine-DNA methyltransferase (MGMT) promoter [6]. MGMT is a DNA repair enzyme that plays a key role in DNA repair and contributes to GBM resistance to treatment. The methylation of the promoter region of MGMT can result in the epigenetic silencing of the MGMT gene and confer TMZ sensitivity [7]. These genetic and epigenetic molecular markers may also be relevant because of their role in autophagy [8]. Despite recent progress, the prognosis for patients with GBM remains poor. There is an urgent need to explore additional therapeutic targets that can be used in combination with current treatments to improve survival and quality of life. Fortunately, recent years have seen significant advances in understanding the mechanisms of tumorigenesis in GBM, with the discovery of new molecular and cellular pathways. Among these, considerable interest has centered on pathways relevant to the aging process, including autophagy and cellular senescence. Various lines of evidence now suggest that both autophagy [3] and cellular senescence [9] are required for GBM development and progression (Figure 2).

This review highlights the interplay between autophagy and cellular senescence in GBM pathogenesis in the context of aging and explores the relevance of understanding these interactions for the development of new therapies for GBM.

## 2. Mechanisms of Oncogenesis in GBM and Its Relationship with Aging

**Figure 3:**
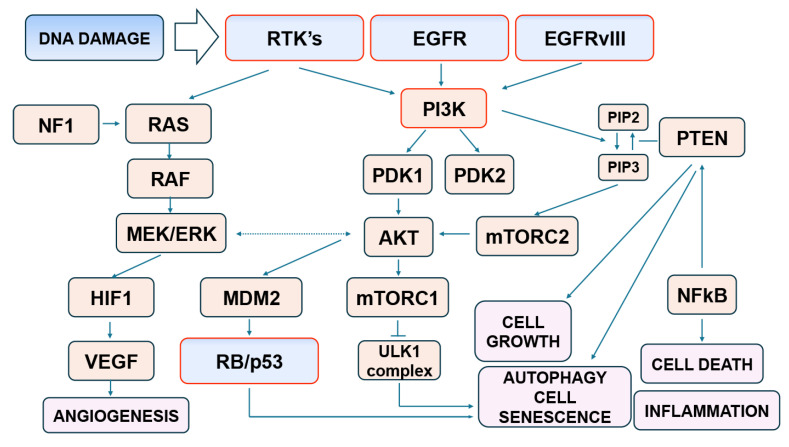
Main signaling pathways involved in GBM pathogenesis. These pathways play a role in cell growth, autophagy, and cell senescence. The RTKs, EGFR, RB/p53, and PI3K/Akt are depicted by a red box. The downstream cellular mechanisms are illustrated in light pink.

While GBM can occur at any age, advanced age is a significant risk factor, and older adults often experience worse survival rates and may be more susceptible to treatment side effects. The typical age at diagnosis is 60 years or older [1]. Aging includes telomere attrition, epigenetic alterations, genomic instability, mitochondrial dysfunction, loss of proteostasis, stem cell exhaustion, altered intercellular communication, dysbiosis, dysregulated nutrient sensing, chronic inflammation, cellular senescence, and alterations in autophagy [2]. It is likely that all these aging-related mechanisms interact and contribute, to varying degrees, to the pathogenesis of GBM. In this review, we will focus on the role of the interplay between autophagy and cellular senescence. Along these lines, a complex interplay between genetic, epigenetic, and environmental factors also plays a role in the pathogenesis of age-dependent GBM (Figure 2). Among them, alterations in signaling pathways, many of them involved in cellular aging processes such as cell growth/survival, senescence, autophagy, and inflammation, have emerged in recent years as important in GBM [3] (Figure 2). The key oncogenic molecular mechanisms involved in these cellular alterations include the following: (i) dysregulation of growth factor signaling involving receptor tyrosine kinases (RTKs) [4]; (ii) activation of the phosphoinositide 3-kinase (PI3K)/AKT/mTOR pathway, and (iii) inactivation of tumor suppressor pathways like p53 and retinoblastoma (RB) [5] (Figure 3). Remarkably, GBM stem cells frequently show the overexpression of growth factors and their receptors, leading to autocrine growth-promoting loops. Specifically, the aberrant epidermal growth factor receptor (EGFR), platelet-derived growth factor receptor (PDGFR), and other RTK signaling pathways are common, with mutations like EGFRvIII frequently observed [6] (Figure 3).

Another important mechanism of oncogenesis in GBM involves mutations and inactivation of tumor suppressor genes, such as p53 [7], RB [8] and PTEN (phosphatase and tensin homolog) [9] (Figure 3). Impairing their ability to regulate cell cycle checkpoints and apoptosis results in abnormal glial cell growth and dedifferentiation. Moreover, phosphatase and tensin homolog (PTEN) interacts with DAXX, and in turn, PTEN directly regulates GBM oncogene expression by modulating DAXX-H3.3 association on the chromatin independently of PTEN enzymatic activity [10]. Additionally, GBM cells often exhibit aberrant and persistent activation of inflammatory and remodeling pathways, like the nuclear factor kappa-light-chain-enhancer of activated B cells (NF-κB) [11] and altered extracellular matrix (ECM) interactions, contributing to tumor growth, invasion, and resistance to therapies. NF-κB is abnormally activated in response to GBM-generated stimuli, resulting in greater cell invasion, promotion of mesenchymal identity, and resistance to radiotherapy [11] (Figure 3). Several mechanisms have been proposed to alter NF-κB signaling in GBM, including EGFR and PDGFR, which are RTKs; these pathways also involve both protein kinase B/AKT (AKT)-dependent and -independent pathways [12,13]. Moreover, stem-like GBM cells exhibiting mesenchymal phenotypes are associated with a more rigid ECM. These cells interact with their microenvironment, which results in the increased accumulation of hyaluronic acid (HA) and tenascin-C (TNC), facilitating tumor migration [14].

**Figure 4:**
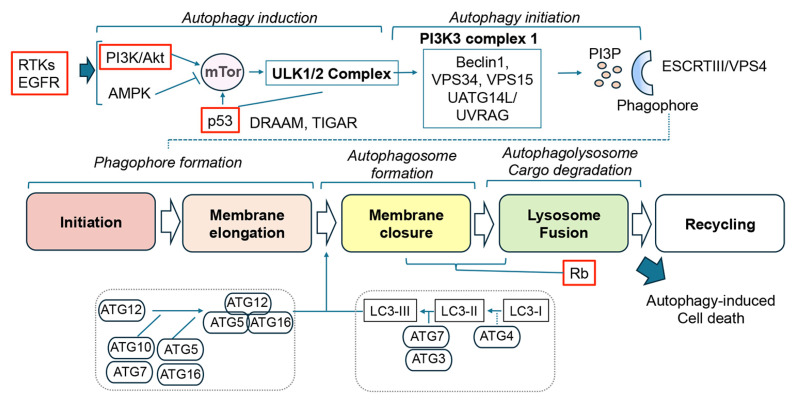
Abbreviated depiction of the macroautophagy pathway. Included are key molecular drivers relevant to GBM pathogenesis (red box) and subcellular steps such as induction, initiation, phagophore, autophagosome formation, degradation and recycling.

Finally, a critical pathway activated by RTKs is the PI3K/Akt/mTor complex, which, under baseline conditions, promotes cell proliferation, survival, and migration and is also remarkably involved in the autophagy pathway (Figure 3). Previous studies have shown that mutations in PTEN result in the de-inhibition and amplification of PI3K/Akt/mTor pathway components, which trigger GBM development and are associated with poor prognosis [10]. Moreover, PI3K/Akt/mTor signaling is downstream of RTKs, such as the PDGFR and EGFR pathways, indicating that the dysregulation of autophagy, a conserved cellular process involved in the degradation and recycling of cellular components, plays an important role in GBM pathogenesis and response to therapy [15]. The PI3K/Akt/mTOR signaling network is activated in almost 90% of all GBM, and PI3K/Akt is a survival signal in GBM [5]. Interestingly, the PI3K/Akt/mTOR pathway is linked to autophagy and plays a crucial role in GBM development and dysregulation (Figure 3). PI3K/Akts is upstream and activates mTor. Specifically, Akt, a downstream regulator of PI3K, directly phosphorylates and activates mTOR, and it can also indirectly activate mTOR by inactivating the tuberous sclerosis complex TORC2, which inhibits mTOR. While mTor activation inhibits autophagy [16], blocking this complex de-inhibits and activates autophagy, with rapamycin being a classical compound that has these effects [17] (Figure 3). Inhibiting this pathway can promote autophagy, which can either promote or inhibit GBM growth depending on the conditions. Understanding this signaling crosstalk is crucial for developing effective targeted therapies for GBM [15] (Figure 4).

In summary, the dysregulation of aging-related mechanisms, such as RTKs, p53, Rb, and PI3K/Akt/mTor, might play an important role in the pathogenesis of GBM via alterations in autophagy and cell senescence.

## 3. Role of Autophagy on GBM Pathogenesis in the Context of Aging

**Figure 5:**
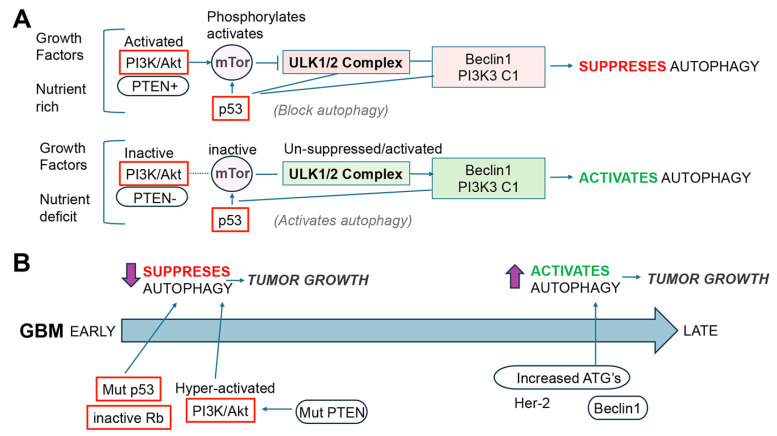
The regulation of the autophagy pathway and the paradoxical relevance to GBM pathogenesis. (**A**) Under baseline conditions, PI3K/Akt phosphorylates mTor, which in turn inhibits the ULK1/2 complex and suppresses autophagy, while inactivation of mTor releases ULK1/2 and activates autophagy. (**B**) At early stages of GBM, mutations and molecular alterations in p53/Rb, RTKs, PTEN, and PI3K/Akt (red box) might suppress autophagy, which in turn could promote tumor growth, while at later stages, alterations in downstream molecular regulators, such as ATGs, activate autophagy and trigger tumor growth. Red indicates inhibition, and green indicates induction.

Autophagy is a critical cellular process that clears damaged or aggregated molecules and organelles via lysosome-dependent degradation to support healthy cellular differentiation, development, and survival [18,19] (Figure 4). While autophagy is closely linked to cellular homeostasis, the relationship among autophagy, aging, and GBM is a critical area of research toward developing new therapeutics. Alterations in the autophagy pathway in cancer have a complex and often paradoxical role, and they are dependent on the microenvironment, stage of tumorigenesis, and initial conditions [6]. In the early stages of carcinogenesis, autophagy is inhibited, and during later stages, it might be stimulated depending on the growth and metabolic needs of the neoplasm (Figure 5). Along these lines, previous studies have shown that for therapeutic purposes in premalignant stages, stimulating autophagy might prevent malignancy [17], while in the late stages of oncogenesis, enhancing or inhibiting autophagy might slow a tumor’s growth [20,21] (Figure 5). Thus, autophagy can promote tumor suppression during cancer initiation and protect tumors during progression. This is why enhancing and blocking autophagy have been proposed as therapeutic strategies in cancer therapy, with the vast majority focused on inhibiting autophagy [22] (Figure 5). As with other types of neoplasms, the relationship between GBM and autophagy is complex, and it could act both as a tumor suppressor and a promoter of survival and progression. This heterogeneous role of autophagy in cancer and gliomas might also be related to the intrinsic complexity of the autophagy process.

There are at least three main types of autophagy, including macroautophagy, microautophagy, and chaperone-mediated autophagy (CMA) [19]. For this review, we will focus on the role of macroautophagy in GBM (Figure 4). However, it is important to consider that microautophagy and CMA also play a role in gliomas and that these mechanisms of autophagy might not operate as exclusively independent processes but are likely co-regulated and act in coordination. Macroautophagy is the best characterized form of autophagy [19]. It involves the formation of a double-membrane-bound vesicle called an autophagosome that engulfs cytoplasmic cargo (Figure 4). The autophagosome then fuses with a lysosome, where the engulfed cargo is degraded [23]. Macroautophagy can be selective or nonselective, depending on the specific cargo being targeted, and it is regulated by signaling pathways, including PI3K/Akt/mTor, AMP-activated protein kinase (AMPK), and p53 [19,24] (Figure 4).

When activated by growth factors and nutrients, mTor inhibits autophagy [25,26]. Conversely, nutrient deprivation and cellular stress activate AMPK, which can inhibit mTOR and thus promote autophagy. Downstream of mTor/mTORC1, the various steps of the macroautophagy process include the following: (i) initiation, (ii) membrane elongation, (iii) membrane closure, (iv) lysosome fusion, and (v) recycling (Figure 4). Each of these stages is regulated by a complex series of molecular steps involving the following: (i) the ULK complex (FIP200, ATG13, 101, and ULK1,2 kinase); (ii) the PI3K3 complex I (VPS34, VPS15, Beclin 1, ATG14, and NRBF2); (iii) ESCRTIII/VPS4; (iv) the complex HOPS/STX7/SNAP29/XKT6; v) SNX17, SNX4, SNX5, and ATG9 [19], respectively. In contrast, p53, a tumor suppressor, can induce autophagy in response to cellular stress, and it can activate genes involved in autophagy, such as DRAM, while also inhibiting other genes that suppress autophagy, like TIGAR [27] (Figure 4).

Interestingly, all three key molecular signaling pathways involved in GBM (e.g., p53, Rb, PI3K/Akt/mTor) [28] also play an important role in aging and autophagy (Figure 5A). For example, in response to stress and age-dependent DNA damage, nuclear p53 directly binds to and activates genes involved in the autophagy process, such as DRAM1 (damage-regulated autophagy modulator), Ulk1, and Atg7. While under non-stress physiological conditions, cytoplasmic p53 inhibits autophagy. In contrast, mutations in p53 that block autophagy contribute to GBM growth by reducing the clearance of misfolded proteins and damaged organelles [29] (Figure 5B). As for RB, studies have shown that silencing this gene can block the final phases of autophagy, specifically the fusion of autophagosomes with lysosomes, leading to an accumulation of undegraded material within the cell. Rb also contributes to regulating the cross-communication between apoptosis and autophagy, favoring GBM resistance to chemotherapy [30] (Figure 5). Moreover, as explained above, PI3K/Akt/mTor carries out regulation by inhibiting macroautophagy, and in GBM, PI3K is overactive due to mutations in PTEN and EGFR [15,31] (Figure 5B).

In GBM, autophagy might be affected at various stages depending on the phase of tumor progression (Figure 5). For instance, Beclin 1 [32], a major component of macroautophagy membrane elongation, has dual roles in promoting survival and inducing cell death, and Beclin 1 plays a complex and context-dependent role in GBM. While it is generally thought of as a tumor suppressor, its expression levels and interactions with other cellular pathways can either promote or inhibit GBM progression [6]. Significant predictive factors for GBM patients have been identified through numerous autophagy-related gene (ATG) signatures, showing that high autophagy scores are linked to an unfavorable prognosis [33]. This study found that most high-risk patients had the IDH wild-type and the non-methylated MGMT promoter, and they had more malignant tumor subtypes (including classical and mesenchymal), predicting poor prognosis. Cox regression analyses further confirmed the independent prognostic value of our signature. Patients classified as high risk using the 14-autophagy gene signature had more malignant characteristics than those classified as low risk [34]. Moreover, increased levels of ATGs have been associated with the aggressiveness of GBM, leading to reduced survival rates and the advancement of tumors [35]. Autophagy can also be associated with GBM progression, where the levels of autophagy molecules such as LC3 and p62 are associated with a poor prognosis [36]. In addition, GBM often exhibits an increased expression of other autophagy-associated proteins with the amplification of ULK1/ULK2 [37] and lysosomal markers such as Cathepsin D and LAMP2A; in addition, the lysosomal biogenesis transcription factor TFEB was found to be increased in GBM [37] (Figure 5).

Other autophagy mechanisms include microautophagy, where the lysosome directly engulfs cytoplasmic cargo and chaperone-mediated autophagy (CMA) [19]. This process relies on chaperone proteins such as HSC70 and the receptor protein LAMP2A on the lysosomal membrane to facilitate the transport of specific proteins across the lysosomal membrane. The targeted proteins are unfolded and degraded within the lysosome [19]. Moreover, CMA is highly specific, targeting proteins with a particular KFERQ motif. Both microautophagy and CMA might also contribute to cancer development; for example, LAMP2A is highly increased in malignant tumors, including GBM, and it is a predictor of poor prognosis [38], while the downregulation of other CMA proteins, such as PHLPP1, has been found in several tumors [39]. Both microautophagy and CMA appear to be also involved in the mechanisms of response of GBM to TMZ treatment [40]. While autophagy can be a protective mechanism for cancer cells, promoting their survival under stress, it can also be exploited as a therapeutic target. In GBM, autophagy can contribute to both tumor cell survival and apoptosis, and its dysregulation is implicated in resistance to therapies like temozolomide (TMZ) [15] (Figure 5).

In summary, all three autophagy mechanisms are dysregulated in GBM, and depending on the context, they can contribute to tumor development and progression. In the early stages of GBM, autophagy mechanisms might be blocked, while at later stages, they might be stimulated (Figure 5B). Macroautophagy has been the most widely studied; however, in microautophagy, CMA also participates, as it is related to the mechanisms of chemotherapy resistance. Molecular mechanisms include the dysregulation of the p53, RB, and PI3K/Akt/mTor pathways, which are also relevant to aging and autophagy. In GBM, alterations of these pathways result in the increased expression of several autophagy molecules that drive malignant transformation and are associated with poor prognosis. In this context, the question is then as follows: which cellular mechanisms are affected by the autophagy alterations that might contribute to GBM pathogenesis and its relationship with aging and cell senescence?

## 4. Macroautophagy-Driven Cellular Mechanisms Involved in GBM Pathogenesis

**Figure 6:**
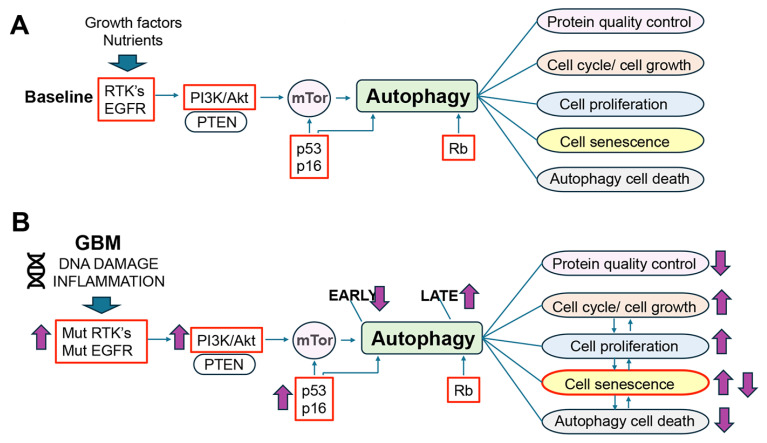
Illustration of potential downstream cellular pathways intersecting with autophagy and their significance to GBM pathogenesis. (**A**) Under physiological conditions, RTKs, PI3K/Akt, and p53/Rb modulate autophagy, which in turn regulates protein quality control, cell cycle/growth, proliferation, senescence, and cell death. (**B**) Under pathological conditions involving environmental factors, genetic alterations, and sustained DNA damage, RTKs, p53/Rb, and PI3K/Akt are hyperactivated, resulting in autophagy suppression during early stages of tumorigenesis, which in turn increases cell proliferation, cell cycle progression, and cell senescence. Arrows show the tendency, with the caveat that the effects are different at early vs. later stages of tumorigenesis.

During aging and in neurological disorders such as Alzheimer’s and Parkinson’s Diseases, alterations in autophagy have been closely linked to the neurodegenerative process [41,42]. In these neurodegenerative disorders, it has been proposed that given the crucial role of autophagy on protein clearance control, alterations in autophagy might lead to the chronic accumulation of proteins such as Abeta, tau, a-synuclein, and TDP43; in combination with other factors, the aggregation of these proteins might be neurotoxic by propagating from cell to cell, triggering inflammation, oxidative stress, and mitochondrial and metabolic alterations [41,43]. In GBM, the effects of the autophagy-dependent alterations in protein quality control in tumorigenesis are less clear. However, autophagy under healthy conditions and during the aging process is not only crucial for protein quality control but also regulates cell survival, cell death, and genomic stability, among other cellular functions [43,44] (Figure 6A).

Autophagy plays a crucial role in maintaining cellular homeostasis by removing damaged organelles and proteins and providing energy during periods of nutrient deprivation [45]. This process can contribute to cell survival by removing components that could disrupt cell division or function (Figure 6A). Alterations in this aspect of autophagy in cell survival could lead to tumor proliferation, including [46] the promotion of autophagy by releasing Beclin 1 from Rubicon, resulting in abnormal cellular proliferation associated with increased autophagy activity. Moreover, in GBM, HER2 overexpression accelerates proliferation and induces autophagy by increasing the protein expression of ATG12, ATG5, ATG7, ULK1, and FIP200, leading to the binding of HER2 to ATG12 [1]. However, as explained before, in GBM, these signaling pathways can both activate and inhibit autophagy, affecting different aspects of oncogenesis in this malignancy (Figure 6A).

In specific situations, autophagy can lead to autophagic cell death (ACD) [47], which is different from other forms of cell death, such as apoptosis (Figure 6A). This can occur under stress conditions or during pathological events like ischemia [47]. Autophagy-associated cell death may act as a tumor suppressor, with several autophagy-related genes deleted in cancers [48]. For example, Beclin1 (also known as ATG6) is deleted in some cancers, including GBM. Other autophagy-related genes, such as ATG5, ATG7, and p62, relevant to ACD, have also been involved in carcinogenesis, with mutations or deletions found in various cancer types [20]. Beclin1 mRNA and protein expression are decreased in GBM; this lower expression has been linked to poor prognosis in GBM patients [49] (Figure 6B).

Moreover, autophagy is implicated in regulating the cell cycle by selectively degrading some of the cell cycle proteins, such as Cyclin D1 and cyclin-dependent kinase 1 (CDK1)-Cyclin A2, influencing cell cycle progression and potentially preventing aberrant cell division [50] (Figure 6A). Besides their well-known role in the cell cycle, it is now clear that CDKs, cyclins, and CKIs play crucial roles in other cellular processes such as transcription, mRNA processing, epigenetic regulation, metabolism, and stem cell renewal [51]. In GBM, alterations in Cyclin D1 and CDK1-Cyclin A2 pathways frequently contribute to tumor proliferation, invasion, and treatment resistance [51]. Furthermore, alterations in the CDK4/6-Cyclin-D-Rb pathway are common in GBM. These alterations often involve deletions and mutations in CDKN2A/B/C and RB1 and, less commonly, genomic amplifications of CDK4, CDK6, and individual D-type cyclins. Also, p18(INK4c), a CDK inhibitor, has been identified as a tumor suppressor gene in GBM, with deletions contributing to tumor pathogenesis [52]. This is why targeting CDK4/6 with inhibitors such as palbociclib, abemaciclib, and ribociclib has been tested in GBM and shown to extend survival by 7–10 weeks [51].

Related to these autophagy-induced cell cycle alterations, GBM neoplasms display considerable genetic instability and frequent DNA double-strand breaks and mutations [53]. Conversely, the DNA damage response signaling in GBM mediates treatment resistance by promoting cell cycle arrest to permit DNA repair and allowing the uncontrolled cell proliferation to continue [54]. Interestingly, alterations in autophagy, cell cycle, and DNA stability are all hallmarks of aging that lead to cell senescence, cell death, and inflammation [2] (Figure 6B). This might help explain why these high-grade gliomas might be more common in aged individuals and how the dysregulation of aging mechanisms might contribute to GBM pathogenesis.

## 5. The Interplay Between Cell Senescence and Autophagy in GBM

**Figure 7:**
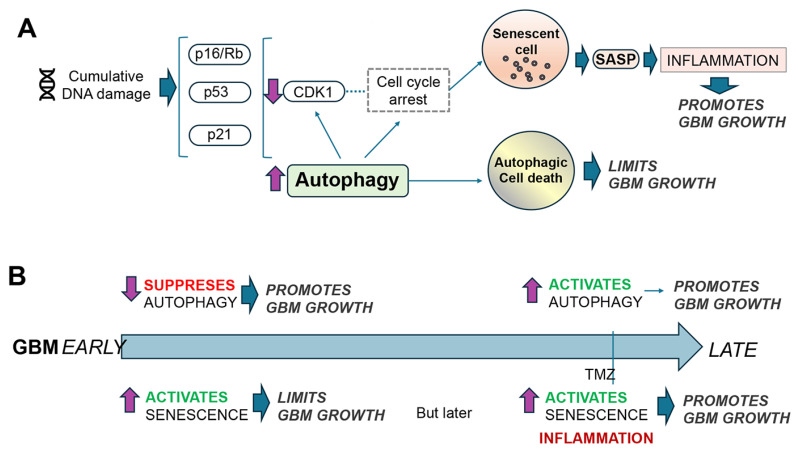
Depiction of some cell cycle mechanisms regulated by autophagy relevant to the complex interplay with cell senescence. (**A**) Age-associated DNA damage is activated via RTKs p16, Rb, p53, and p21, resulting in increased recycling of CDK1 via autophagy, which in turn arrests the cell cycle and could result either in cell senescence or cell death. (**B**) In the early stages of GBM, autophagy can be suppressed, which in turn activates cell senescence. In the later stages, cellular senescence, along with the release of SASP factors and inflammation, can promote tumor progression. Red indicates inhibition, and green indicates induction.

Cellular senescence is an aging-associated state of cell cycle arrest, and if senescent cells accumulate progressively, they can also play a role in aging-related diseases [55]. DNA damage during aging triggered by stress, radiation, chemicals, and other environmental toxins is a major contributor to cellular senescence (Figure 6B and Figure 7A). When DNA damage is cumulative, cells may either undergo apoptosis or enter a senescent state. Signaling via p16/Rb, p53, and p21 is crucial for cell cycle arrest and senescence [56] (Figure 7A). Remarkably, as described before, these transduction pathways are also involved in autophagy [57] and GBM development [7] (Figure 3). Senescent cells can promote a chronic inflammatory state, which is a characteristic of aging tissues and is associated with various age-related diseases. During this process, senescent cells display the presence of secretory granules known as senescence-associated secretory phenotypes (SASPs), which express proinflammatory cytokines and chemokines [58,59] (Figure 7A). Cell senescence is an important factor in GBM, both as a potential tumor suppressor mechanism and a contributor to tumor recurrence and progression [60] (Figure 7A). High-grade gliomas can be induced into senescence by treatments like radiation and temozolomide (a guanine alkylating agent), but they also possess mechanisms to evade this state [61] (Figure 7B).

The occurrence of senescence in GBM after treatment can also potentially lead to disease recurrence due to the release of proinflammatory and mitogenic cytokines in the surrounding microenvironment [58] (Figure 7B). The inhibition of autophagy has been shown to work together with other chemotherapeutic agents in inducing SASP [62]. Furthermore, glial stem cells (GSCs) remaining in brain tissue after radiation and chemotherapy become chemoresistant, leading to accelerated tumor growth [63]. A recent study showed that inhibiting O-GlcNAcylation in GBMs decreases cell viability, inhibits autophagy, and increases sensitivity to TMZ [64].

Autophagy can help remove damaged mitochondria and proteins, which can contribute to senescence (Figure 7). By clearing these components, autophagy can help maintain cellular health and delay the onset of senescence [65]. For this reason, in the initial state, the activation of autophagy could be protective, and it suppresses senescence as it helps remove damaged organelles and proteins. The sustained activation of autophagy can promote senescence by facilitating the synthesis of SASP proteins [66] (Figure 7A). Autophagy is activated during oncogene-induced senescence, and the knockdown of several key autophagy regulators delays oncogene-induced senescence, especially the SASP (Figure 7A). In fact, the TOR–autophagy spatial coupling compartment (TASCC) is involved in protein synthesis of some SASP factors [66]. At the TASCC, autophagy provides amino acids to activate mTOR, which in turn promotes the synthesis of proinflammatory cytokines, such as IL6 and IL8. Thus, the TASCC connects the cell’s catabolic (autophagy) process to its anabolic (protein synthesis) process for efficiently coordinating the synthesis of SASP proteins during oncogene-induced senescence [66]. For example, the overexpression of CDK inhibitors such as p16, p19, and p21 activates autophagy and cellular senescence in both human fibroblasts and breast cancer cells [66,67]. In contrast, the autophagy of KEAP1 promotes redox homeostasis during senescence, and the selective autophagy of TNIP1 enhances inflammation [68], which plays an important role in reactivating tumorigenesis in GBM.

Finally, it is important to emphasize that, as with autophagy, cellular senescence in GBM presents a complex scenario (Figure 7B). While at first acting as a hurdle to tumor growth, the long-term presence of senescent cells, particularly after therapy, can generate an inflammatory pro-tumorigenic microenvironment that facilitates GBM recurrence and progression (Figure 7B). Therefore, targeting senescent cells with senolytic agents aimed at ameliorating the consequences of SASP is a potential new therapy for the management of GBM [61] (Figure 7B).

## 6. Discussion and Conclusions

Aging is the most important risk factor for several disorders of the CNS, including gliomas such as GBM, neurodegeneration, and cerebrovascular disease, among other major morbidities affecting the US population [69,70,71]. Understanding how the dysfunction of the interplay between aging-relevant mechanisms, such as autophagy, cell senescence, and cell cycle, takes place is key to identifying novel targets and therapeutics for high-grade gliomas. Although this manuscript focuses on autophagy and cell senescence interactions in GBM, the contribution of other hallmarks of aging, such as DNA damage, mitochondrial dysfunction, proteostasis alterations, stem cell alterations, and inflammation, needs to be taken into consideration. A major obstacle in developing new GBM therapies that target these aging mechanisms is the paradoxical and stage-dependent effects, which are influenced by the microenvironment. For instance, at early stages of GBM, autophagy mechanisms might be blocked, while at later stages, they might be stimulated [72] (Figure 5 and Figure 6). As for cell senescence and GBM, at early stages of tumorigenesis, this process might impede tumor expansion; however, the chronic accumulation of senescent cells promotes a proinflammatory microenvironment that facilitates GBM recurrence and progression [55] (Figure 7).

Autophagy seems to be suppressive of GBM growth during the early stages of tumorigenesis, while at later stages, it might be tumor-promoting [68]. Thus, it will appear that non-specifically stimulating autophagy early and blocking autophagy at later stages of GBM might be a potential strategy (Figure 6). However, it is worth considering the following: (i) There are at least three main autophagy mechanisms that crosstalk; (ii) for macroautophagy, there are several entry points (e.g., initiation, membrane elongation, membrane closure, lysosome fusion, recycling); (iii) hundreds of proteins are involved in coordinating and regulating autophagy, making it difficult to determine which targets are the best. Moreover, autophagy plays a complex and multifaceted role in cell senescence [68]. Then, if autophagy is activated therapeutically at early stages of GBM, one possible negative consequence is that it can block cell senescence, which is needed as a defense mechanism to limit the growth of the tumor (Figure 6). Conversely, autophagy activation can also promote cell senescence by facilitating the synthesis of SASP [66], and selective autophagy regulates senescence, with specific autophagy pathways targeting proteins involved in redox homeostasis, translation, and inflammation [73] (Figure 7). Furthermore, blocking autophagy can make GBM cells more sensitive to chemotherapy and radiation, potentially reversing treatment resistance [74] (Figure 6). On the other hand, promoting autophagy can trigger cell death; for instance, natural compounds and mTOR inhibitors can activate autophagy, potentially leading to cell death in GBM cells [74]. Depending on the stage and context, this may induce cell senescence, potentially encouraging further tumor growth (Figure 6).

Recent trials have aimed to block autophagy to increase GBM sensitivity to TMZ and radiation. Examples of drugs inhibiting autophagy that have been tested alone or in combination in GBM include chloroquine and hydroxychloroquine [3,75], but the results have not been consistent [75]. The problem with these currently available autophagy inhibitors is that they lack specificity and may have side effects and toxicity when combined with other drugs [76]. Other trials have investigated the effects of promoting autophagy with mTor inhibitors, including itraconazole/icaritin [77], terosirolimus, or ABTL0812. In the case of terosirolimus, it has been tested alone or in combination with other drugs like perofosine and bevacizumab. While some studies report promising results, others report limited efficacy [78]. Clinical trial results for autophagy inhibitors in GBM have been mixed [79]. In the case of ABTL0812, this oral drug is currently in phase II trials. It inhibits the mTor/PI3K/Akt pathway, resulting in cell death through autophagy and has shown synergistic effects with radiotherapy and TMZ in preclinical studies [72]. Novel autophagy drugs are being investigated in GBM, either alone or in combination with other treatments. Some drugs, like trifluoperazine [80], are being investigated for their capacity to block autophagy and increase radiosensitivity. Other approaches focus on modulating autophagy through targeting pathways like mTOR or STAT3, often in combination with other therapies [81]. In this regard, immunotherapy with CAR-T cells and mRNA vaccines has shown promise in the management of GBM. This is important because they will need to be taken into account in future combinatorial therapies, as modulating cell senescence and autophagy also plays a very important role in regulating immune responses.

Targeting cell senescence in GBM has also been explored using senolytic drug combinations aimed at ameliorating the consequences of SASP at later phases of the disease or post-radiation and post-chemotherapy [61]. Research has identified specific pathways and targets involved in GBM senescence, including NRF2, Bcl-xl, clAP2, and STAT3, which can be engaged with senolytic drugs [82]. Other senolytic drugs have also been tested as anti-aging compounds, such as combinations of quercetin (flavonoid) with dasatinib (tyrosine receptor inhibitor) and navitoclax (Bcl2 protein inhibitor) [83]; these might be suitable treatments for GBM. These drugs have been shown to reduce the accumulation of senescent cells in models of aging and degenerative disorders [83]. Clinical trials are underway to ascertain the safety and efficacy of senolytic drugs in GBM patients, including those with recurrent or newly diagnosed tumors [61].

Taken together, these studies suggest that the ideal drug or combination of therapies (e.g., small molecule, gene therapy, immunotherapy) will need to block the selective aspects of autophagy relevant to the specific mechanisms of the early stages of GBM progression while minimally affecting cell senescence (Figure 6 and Figure 7). Conversely, at later stages of tumorigenesis or post-treatment, the selective activation of autophagy pathways and anti-inflammatory therapies targeting SASPs will be needed (Figure 6 and Figure 7). Characterizing early and late-stage pathways in GBM models with multi-omics methods (e.g., single-cell nuclear RNA, spatial transcriptomics, proteomics) and computational modeling could help identify the key regulators of autophagy and cell senescence in GBM, similarly to the approaches used by the Accelerating Medicines Partnership-Alzheimer Disease (AMP-AD) consortium in neurodegeneration studies. Finally, for clinical development, we will require well-characterized and validated biomarkers for both autophagy and cell senescence to effectively monitor the effects of these new drug classes. These biomarkers will help identify optimal combinations and specific aspects of autophagy and cell senescence to target in GBM, as well as the appropriate timing, in order to maximize therapeutic outcomes.

## Data Availability

Not applicable.
